# ZincBind—the database of zinc binding sites

**DOI:** 10.1093/database/baz006

**Published:** 2019-02-05

**Authors:** Sam M Ireland, Andrew C R Martin

**Affiliations:** Institute of Structural and Molecular Biology, Division of Biosciences, University College London, Darwin Building, Gower Street, London, UK

## Abstract

Zinc is one of the most important biologically active metals. Ten per cent of the human genome is thought to encode a zinc binding protein and its uses encompass catalysis, structural stability, gene expression and immunity. At present, there is no specific resource devoted to identifying and presenting all currently known zinc binding sites. Here we present ZincBind, a database of zinc binding sites and its web front-end. Using the structural data in the Protein Data Bank, ZincBind identifies every instance of zinc binding to a protein, identifies its binding site and clusters sites based on 90% sequence identity. There are currently 24 992 binding sites, clustered into 7489 unique sites. The data are available over the web where they can be browsed and downloaded, and via a REST API. ZincBind is regularly updated and will continue to be updated with new data and features.

## Introduction

The importance of zinc in living organisms is well established. Since the initial demonstrations that zinc-deficient diets were associated with specific, anaemic conditions in the early 1960s (1), there have been many studies demonstrating the nutritional necessity of dietary zinc to human health (2). At the molecular level, zinc was first shown to be an essential co-factor for a specific enzyme in 1940, when carbonic anhydrase activity was found to be proportional to zinc concentration (3). The precise mechanisms by which zinc enables specific proteins to carry out their functions remained relatively unknown until the first crystal structures of zinc enzymes started being published, beginning with carbonic anhydrase C in 1972 (4).

As the number of zinc protein structures began to grow, attempts began to identify the distinguishing features of the zinc binding sites that mediated zinc affinity in these proteins. The tendency for histidine and cysteine to act as the liganding residues was observed in 1985 (5). Another early characterization was the distinction between catalytic zinc binding sites and structural zinc binding sites (6)—for example, that catalytic sites are almost invariably liganded by one water molecule and three protein residues, while four sulphur ligands predominate in structural sites.

The first systematic attempt to identify all zinc binding sites in the Protein Data Bank in order to identify their distinguishing features came at the end of the 1990s (7), in which 387 zinc-containing structures were obtained in order to identify properties of their liganding shells and the importance of hydrogen bonding. This was followed by a number of other similar studies with increasing numbers of proteins (8–20) with a more recent paper (21) surveying 8474 zinc-containing structures to investigate typical metal-ligand distances. This paper offers a critique of many of the previous papers, in particular noting that many previously identified zinc binding sites were misinterpretations caused by failure to take symmetry into account, and by mislabelling surface zincs as catalytically or structurally important.

While similar criteria were often applied when identifying zinc binding sites (e.g. filtering by 90% sequence identity and by crystallographic resolution and discarding zinc atoms thought to be zinc salts), this was often done manually or using algorithms for which no details were provided. In none of the above cases were the data or algorithms made available electronically for future research.

Three electronic resources have been made available: (i) MESPEUS (22) a zinc database published in 2008, but which is no longer maintained, (ii) ZifBase (23) which is specifically for zinc fingers and (iii) MetalPDB (24, 25), a database of all metal binding sites (including zinc). However, the binding sites in MetalPDB are derived from asymmetric units rather than biologically relevant assemblies.

Here we present ZincBind, an attempt to provide a single central database of zinc binding sites. Unlike MetalPDB, it accounts for biological assemblies. This is particularly important for zinc binding sites since these are often at the interface between copies of a chain. For example, insulin is a hexamer, formed of two trimers, each of which coodinates a zinc. Many structures of insulin (such as PDB entry 1ZEH) provide only one subunit of the insulin hexamer and the asymmetric unit shows only one residue binding to the zinc atom, which is clearly not biologically correct. ZincBind is regularly updated and contains all known zinc binding sites from the Protein Data Bank while automatically discounting zincs that are merely present as part of a salt. The code is open source (github.com/samirelanduk/zincbind), allowing others to maintain their own local repository if they so wish.

## Methods

### Obtaining zinc binding sites

Structures containing zinc were obtained from the RCSB (26) using web services by using a ChemCompFormulaQuery query for the formula Zn. The code iterates through the structures using the Python molecular structure and parser library, atomium (github.com/samirelanduk/atomium/), to process the PDB files. Atomium automatically generates the biological assembly having the lowest energy while still containing zinc by using the assembly instructions given in the PDB file.

From the biological assembly, all metals (zinc and otherwise) are examined and the coordinating residues are identified—in this case non-carbon, non-hydrogen atoms within 3Å (centre-to-centre) of the metal, which do not have a bond angle of less than 45$^\circ $ with a closer liganding atom in that residue. The metals are then clustered such that any metal that shares a liganding residue with another metal is in the same cluster. Metals duplicated because they lie on a point of symmetry rotation have their duplicates removed. Clusters that do not contain a zinc (i.e. metal binding sites that are not zinc binding sites) are removed and the remainder are saved to an SQLite database (www.sqlite.org). Note that this will include multi-metal binding sites (where at least one metal is a zinc) as well as pure zinc-binding sites. However, if two clusters are identical—that is they have the same atom, residue and chain IDs owing to being duplicated when the biological assembly was generated, only one copy is added to the database and a counter indicating how many copies there are of the site is incremented. The chains that contain the liganding residues are also stored, along with their sequence, for the purposes of the clustering steps outlined below.

Some zinc atoms in a PDB file are not identified as part of a binding site, but are stored in the database anyway so as to hold at least some information on every zinc atom. For example, zinc atoms are identified as being part of a binding site only if they have at least two protein liganding residues, and at least three protein liganding atoms (with liganding atoms defined as above). If these conditions are not met, they are presumed to be a salt with no physiological significance. Other reasons for not using a zinc atom are if the PDB file that contains it has only alpha carbons and so cannot be used to identify liganding residues, or if the asymmetric unit contains multiple copies of a chain and the atom is part of a chain that is not included in the biological assembly used.

### Removing redundancy

The next step is to identify redundancy amongst the zinc binding sites. For example, there are several hundred structures of carbonic anhydrase in the Protein Data Bank. Chains in the database are clustered at 90% sequence identity using CD-HIT (27), and the clusters are saved to the database. The binding sites themselves are then clustered such that two binding sites are members of the same cluster if (i) they are associated with the same 90% chain cluster(s) (as created in the previous step), (ii) they have the same residue types in the same order on that chain and (iii) the residues sequentially neighbouring the liganding residues have the same amino acid types.

### The web interface

BLAST database files are created using the NCBI binaries (28) and the finished database is integrated into a web application written using the Python web framework Django V2.1 (djangoproject.com). The binding sites are visualized in the web front-end using the NGL protein viewer (29), and BLAST searching is performed by the NCBI blastp binary. The web REST API is created using the Django REST Framework (django-rest-framework.org).

## Results and discussion

### Prevalence of zinc

Of the 146 856 structures contained in the Protein Data Bank at the time of the last database build in December 2018, 14 099 (9.60%) were found to contain at least one zinc atom according to the search criteria given above. This is remarkably close to the 10% figure that is usually given as the estimated proportion of the human genome thought to utilize zinc (12).

### Qualifying atoms

In total, when run in December 2018, ZincBind identifies 41 789 zinc atoms and 1249 other metal atoms associated with zinc atoms when searching the raw coordinates of the PDB asymmetric units. Other metals are only stored in ZincBind if they are part of a multi-metal binding site with at least one zinc atom, so the vast majority of metals in the database are zinc. Of the non-zinc metals, the most common of these ‘co-active’ metals are magnesium, potassium, iron and copper (see Table 1).

**Table 1:** Frequency of co-active metals in all zinc binding sites. ^*^The count for zinc here refers to the number of ‘extra’ zinc atoms in multi-metal binding sites. Consequently, a binding site with three zinc atoms would contribute +2 to this count. The third column shows the number of zinc-binding sites which contain the metal in question. ^†^This is the number of zinc-binding sites containing more than one zinc atom.

Metal	Count	Site count
Zn	2437^*^	2117^†^
Mg	443	439
K	223	220
Fe	178	143
Cu	128	122
Mn	81	79
Na	74	67
Ca	51	49
Cd	24	23
Co	13	13
Other	8	5

Of the 41 789 zinc atoms in the database, 14 360 (34.3%) are not associated with any binding site, and are in the database solely to acknowledge their existence (see Table 2). The most common reason for not assigning a zinc atom to a binding site is that the atom is duplicated multiple times in the asymmetric unit, but appears only once in the biological assembly used for processing—9691 metals were excluded for this reason. For example, PDB entry 1A4L contains four chains, each with one zinc atom, but the biological assembly only uses one. The other three are stored in ZincBind with an omission reason, but have no binding site assigned to them.

**Table 2:** Reasons for excluding zinc atoms identified in PDB files from being part of a zinc binding site. Here ‘Not in the biological assembly’ generally refers to cases where the asymmetric unit contains multiple identical copies of a chain, and only one is used in the biological assembly, so the zinc atoms associated with the other chains are not processed. Zinc atoms are also rejected if they have one or zero liganding protein residues (‘Too few liganding residues’), or if they have fewer than three liganding protein atoms (‘Too few liganding atoms’). Consequently, a zinc can have only two liganding residues and still be part of a biologically relevant zinc binding site if one of those residues provides two liganding atoms. ‘No side chain information’ refers to PDB files that contain only C $\alpha $ or backbone atoms, and thus cannot be used to identify binding sites.

Exclusion reason	Count
Not in the biological assembly	9691
Too few liganding residues	3819
Too few liganding atoms	827
No side chain information	23

Using the full biological assembly leads to zinc ions being both excluded and included as being associated with binding sites. As stated above, it is common for the asymmetric unit of a PDB file to contain multiple copies of the biomolecule of interest as a result of crystallization, but once the ‘correct’ assembly is chosen, these duplicated zinc atoms will be absent from the final structure. Conversely, using the full biological assembly rather than just the raw asymmetric unit coordinates of the PDB is crucial when the zinc atom is present at an interface between chains. In the asymmetric unit, there may be a single residue coordinating with the ion—if symmetry were not considered, such a model would be discarded by the ZincBind algorithm as a salt.

### Zinc binding sites with high representation

**Figure 1:**
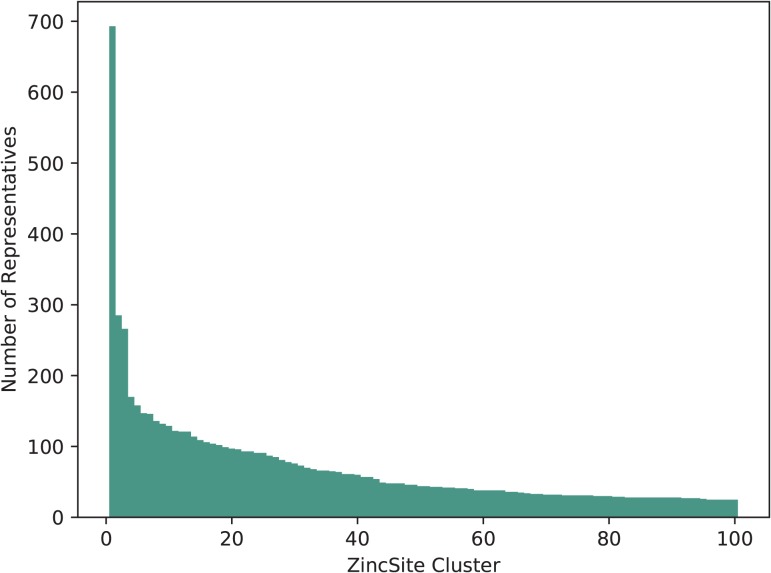
The number of representatives in the largest 100 binding site clusters, ranked on the $x$-axis by the number of sites in that cluster. Carbonic anhydrase, ranked first, can be seen as a clear outlier—it is very highly represented.

The 28 667 metal atoms, for which binding site information is stored, are part of a total of 24 992 zinc binding sites in ZincBind. After clustering the proteins at 90% sequence identity and then clustering the binding sites as described above, there are 7489 unique zinc binding sites in the database. The binding site with the most copies is from carbonic anhydrase (693 copies), a catalytic serum protein and, as already noted, the first known zinc binding protein. This is followed by a nitric oxide synthase zinc binding site (285 copies), an interface site between two chains, and then JMJD2D (266 copies), a lysine-specific demethylase. Figure 1 shows the distribution of binding sites in the largest 100 clusters. A total of 4223 zinc binding sites are currently unique in that they have only one structure.

For each cluster of equivalent zinc binding sites, the best resolution structure is chosen to provide a single site that is flagged as the ‘representative’ for that cluster.

### Liganding residues

The binding residues that dominate zinc binding sites are well established: cysteine, histidine and the acidic residues aspartate and glutamate (7). This is supported by the data in ZincBind. There are a total of 109 363 liganding residues in ZincBind, of which those four residue types, together with water, comprise 92.6% of all zinc-liganding residues (see Table 3). Note, however, that this considers every binding site in the database. When non-redundant sites are studied (i.e. only one site per cluster thereby removing the bias towards more intensely studied proteins), these five comprise a slightly smaller percentage of zinc-liganding residues (91.1%). This suggests that the binding sites most frequently appearing in the PDB show slightly less variation than a more representative sampling.

**Table 3:** Frequencies of the most common zinc-liganding residues (including water) in all binding sites and in representative (unique) binding sites

Residue	Count	
	All	Unique
Cysteine	43 945	11 195
Histidine	31 788	9077
Water	10 179	3668
Aspartate	9124	3107
Glutamate	6238	2472
Other	8089	2519

If the binding residues’ single letter codes are combined to give an overall ‘signature’ of the binding site, C4 (four cysteine residues) is the most prevalent, with 1129 of the 7489 unique binding sites having this arrangement of residues—followed closely by C3H1, with 931. That the most common signature makes up just 15.1% of the total is indicative of the relatively high diversity in such signatures. Between them, the top ten residue signatures account for just 57.6% of the total. The complete distribution of residue signatures is provided in Supplementary File ResidueSignatures.csv.

Of the 21 810 redundant binding sites that contain just one zinc atom, and that come from structures with resolutions better than 3Å, the most common mode of coordination is via four liganding atoms: 14 702 examples (67.4%). 3-coordination and 5-coordination have similar prevalences: 2027 (9.4%) and 3274 (15.2%) respectively. See Table 4 for more details.

**Table 4:** Frequencies of the coordination modes in all single-zinc binding sites with a resolution better than 3Å. Note that the coordination mode includes coordinating waters and consequently there may be some examples where waters are involved in coordination, but are not included in the PDB file—in these cases the coordination mode shown will be one lower than the true count. Consequently these figures should be taken as representative of the distribution rather than absolute numbers.

Coordination mode	Count
Three	2027
Four	14 702
Five	3274
Six	1567
Higher	240

**Figure 2:**
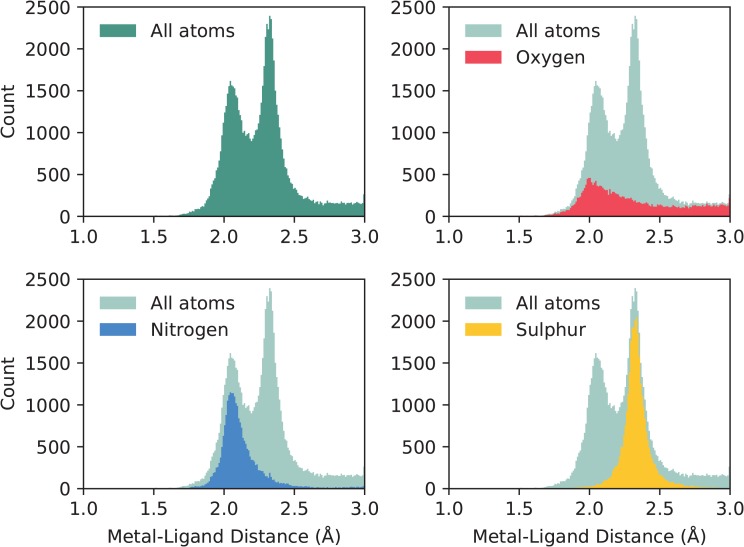
The distribution of liganding atom distances in all single-zinc sites, with a resolution better than 3Å. The overall distribution can be seen as bimodal, with the first peak being the preferred distance of nitrogen and oxygen, and the second peak being the preferred distance of sulphur, which has a greater Van der Waals radius. While nitrogen and sulphur both have tight distributions, oxygen does not, and has a much more prominent tail in its distribution.

This same subset of binding sites can be used to investigate liganding atom distances. Dokmanić *et al.* (15) have suggested that evaluation of geometry is best performed on a full dataset since ‘redundancy is not critical for precise evaluation of the geometrical factors, but high resolution and a large number of events are necessary’. We have followed the same argument and calculated across the full set of structures with resolution better than 3Å. Nitrogen and sulphur atoms both have characteristic distances with tight distributions: $2.12\pm 0.19$Å and $2.33\pm 0.12$Å, respectively. Oxygen, however, has a much wider distribution, as it can be provided by either of the carboxylate oxygens of the acidic side chains, or from water. Its average distance to zinc is $2.31\pm 0.54$Å. The full distribution of liganding atom distances can be seen in Figure 2. We note that when performed on the non-redundant set, the values are similar—N: $2.14\pm 0.23$Å; S: $2.33\pm 0.13$Å; O: $2.34\pm 0.75$Å.

### Co-active binding sites

**Figure 3:**
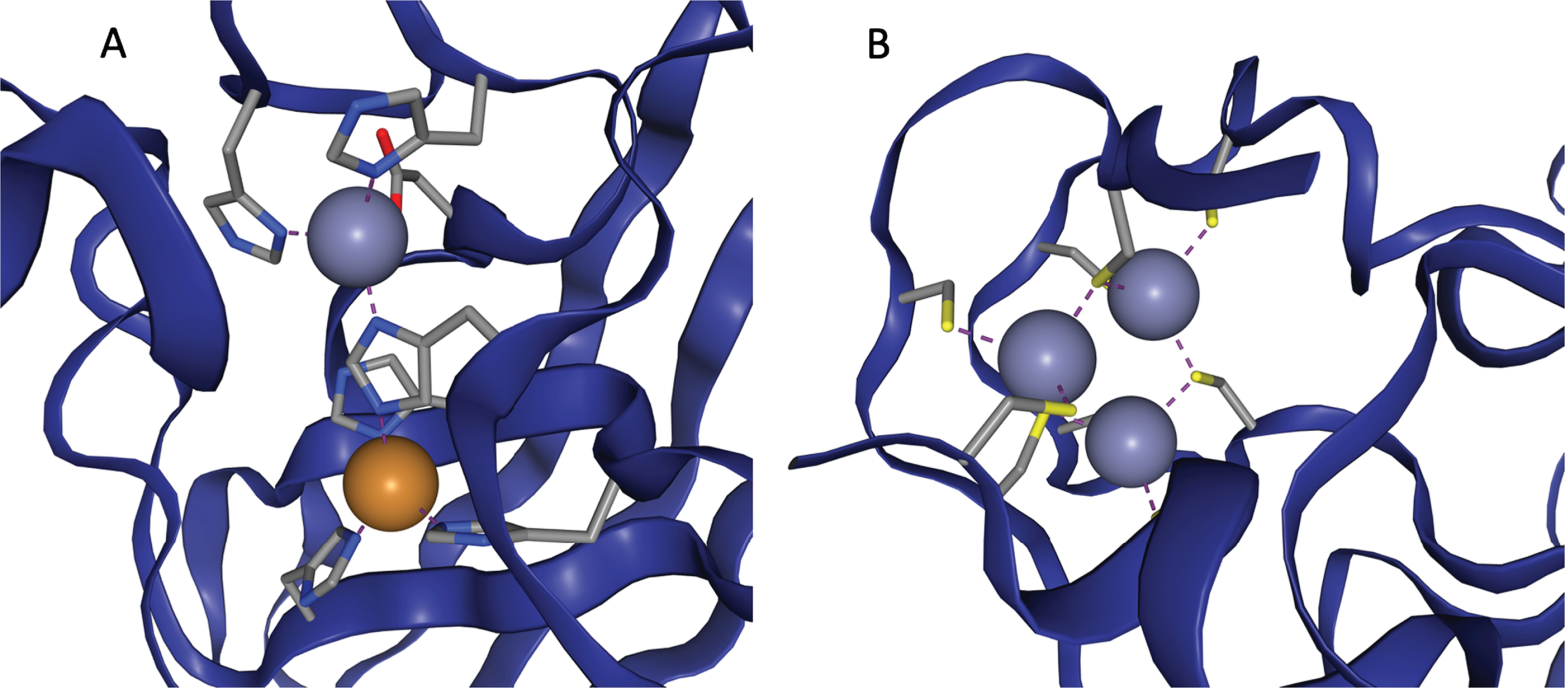
(a) The active site of Cu,Zn Superoxide Dismutase (PDB entry 2SOD). Here a single histidine residue links a zinc atom and a copper atom into a single functional unit. (b) A three zinc co-active site taken from PDB entry 6A5K. Both images are taken from ZincBind.

In some cases multiple metals act in concert to form a single functional unit. Such sites are generally referred to as co-active binding sites in the literature (or ‘co-catalytic’ where the site is known to have a catalytic function). Here such sites are defined as those instances where a single residue is liganded to more than one metal, according to the criteria defined above. The metals are grouped into a single site as described in the methods. A catalytic and non-catalytic example can be seen in Figure 3.

In ZincBind, 3182 from the total of 24 992 zinc binding sites (12.7%) contain multiple metals—2754 contain two metals, 379 contain three, 36 contain four, 10 contain five, and 3 zinc binding sites contain six metals—though two of these are from a synthetic construct.

These multi-metal sites account for all of the non-zinc metals in the ZincBind database. While the term ‘co-catalytic’ is sometimes used interchangeably with ‘co-active’, only 2661 of the multi-metal sites (83.6%) are derived from an enzymatic protein (defined here as a protein name ending in *-ase*). However, this compares with just 61.1% of all zinc-only binding sites that are enzymatic. A Fisher exact test showed the difference to be significant ($P<0.00001$).

### Web interface

**Figure 4:**
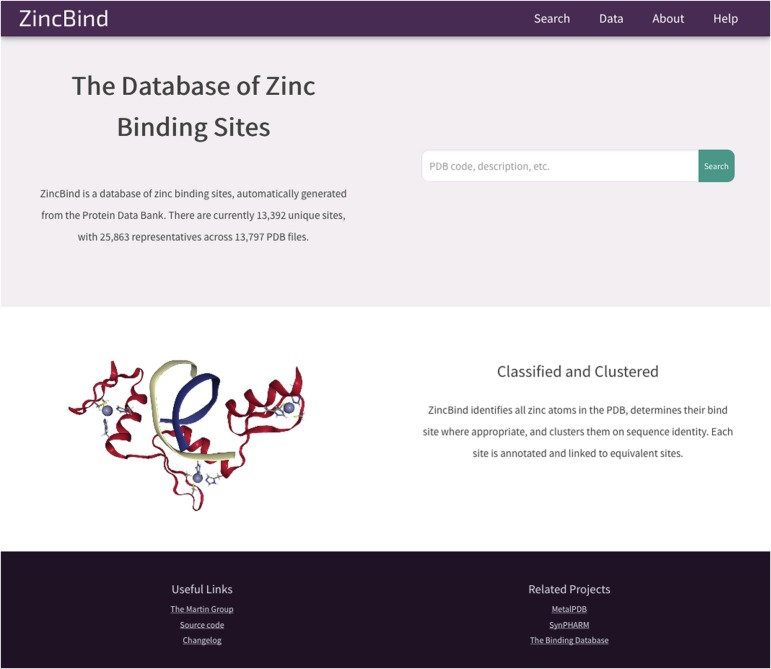
The ZincBind home page. From here the user has the option of performing a ‘quick search’ of the entire database, without having to construct an advanced query on the advanced search page. The links in the navigation bar at the top offer quick access to this search functionality, as well as overviews of the data, browse options and help resources.

The data described in this paper are available via a web interface at the address zincbind.bioinf.org.uk. The ZincBind resource is an open-source Django/Python web application. Figure 4 shows the ZincBind home page.

**Figure 5:**
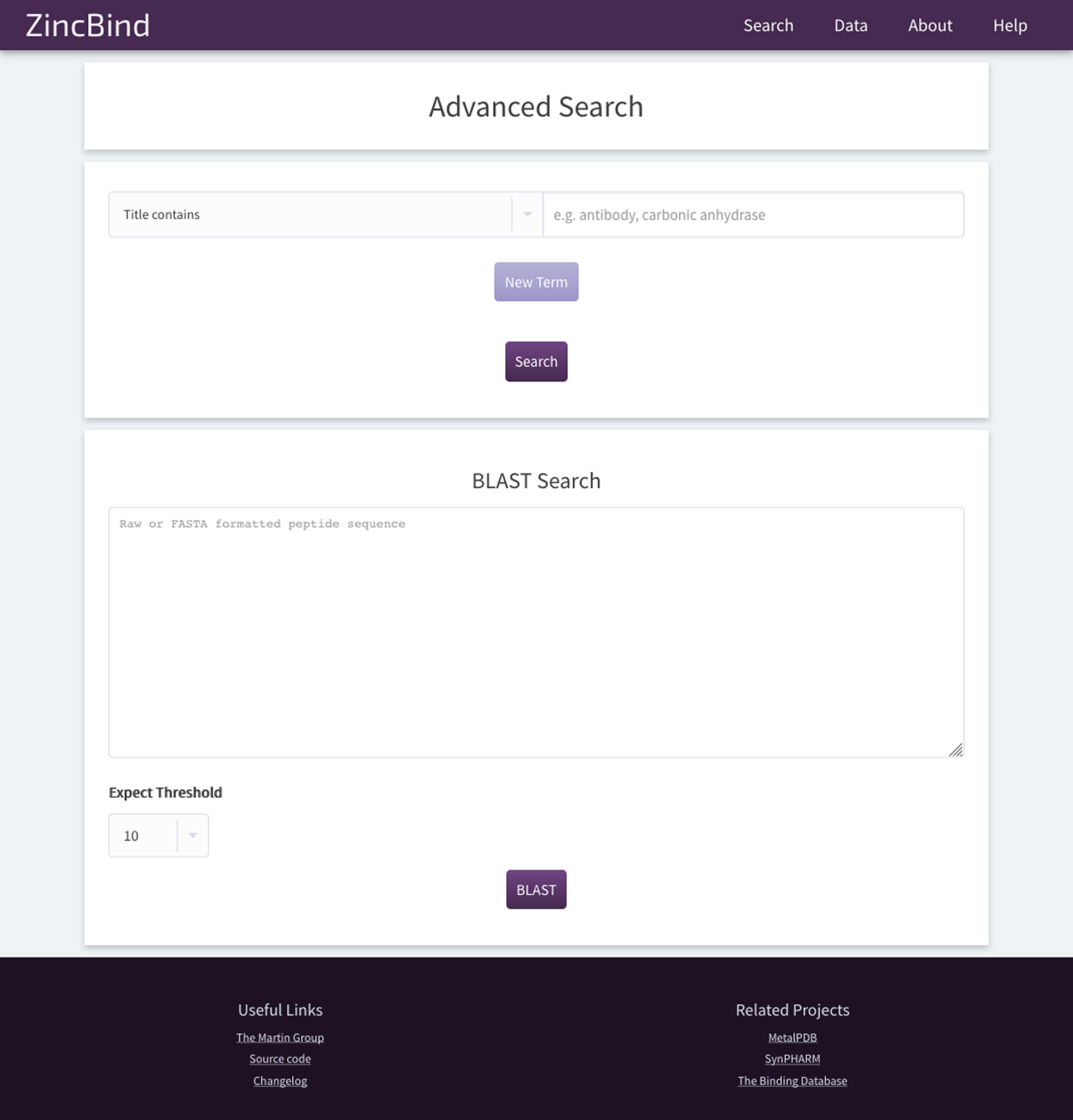
The ZincBind search page. This allows the user to search the database by a particular property, such as PDB title or resolution limit, and also offers the option to BLAST search the zinc-bearing chains in the database. On the resultant search page, there are options to sort the returned results by a number of metrics.

The most recent version at time of writing, version 0.6.2, allows for the data to be searched and sorted by multiple criteria from the search page (Figure 5), or alternatively allows the user to provide a single term in an ‘omni search’ box that searches for the term in multiple fields. The data are returned as a list of PDB files, with the zinc binding sites appearing beneath them.

In addition, the user may search the database by sequence using a BLAST search interface. This uses the sequences of the chains that actually contain the zinc binding residues, not simply all the chains contained in every relevant PDB file. The database contains all relevant PDB files irrespective of resolution; previous attempts to collect zinc binding sites have frequently only included structures with a resolution better than a given cutoff. ZincBind allows a resolution filter to be applied in queries via the web interface, or via web services, allowing the user to exclude sites that do not meet their quality criteria.

Increasingly important in modern bioinformatics is the ability to access and query the data programmatically, so that they can function as part of a larger pipeline. Consequently, ZincBind provides a REST API so that all objects in the database can be accessed as JSON objects and iterated over using the relevant, documented URLs. The user can also search the database using this API. The full database can also be downloaded in either JSON or SQLite format, to permit offline analysis of the data. Indeed, as the repository is open source and contains all the necessary scripts to generate the database, users can run the ZincBind software locally and generate the data using the same algorithm if they so wish.

**Figure 6:**
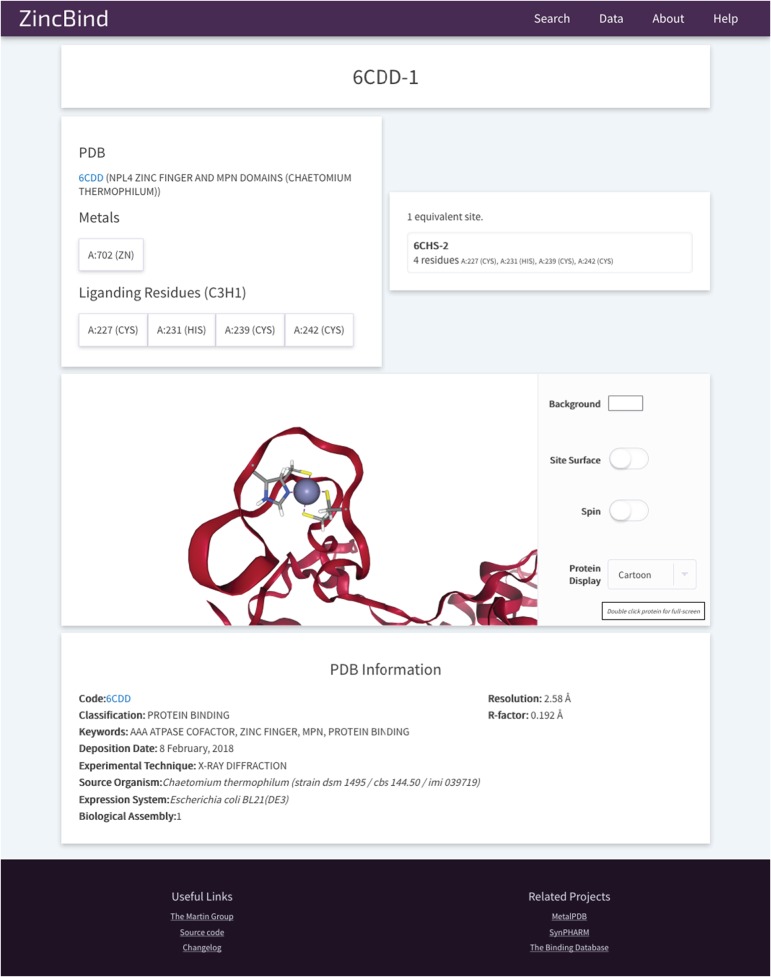
An example of a Zinc Binding Site on ZincBind. This provides an overview of the liganding residues, and the zinc atom(s) in this site. There are links to any equivalent sites that might exist in the database. Below this is a 3D, fully manipulable representation of the binding site, with customizable display options. Finally, there is a summary of the PDB structure from which the site is taken.

The site also contains 3D visualizations of all zinc binding sites (see Figure 6 for an example from PDB entry 6CDD) as well as the structures that contain them, a help/FAQ page, overviews of the data in the form of charts, and a changelog page detailing changes made at each version increment.

## Conclusions

We have created ZincBind, a resource that identifies, collates and presents all known zinc binding sites from the Protein Data Bank. Unlike previous collections of zinc-binding data, the resource is updated regularly. It provides the data in multiple formats, and clusters sites on the basis of 90% sequence identity of the protein chains and matching liganding residue, selecting the highest resolution structure as the representative site. ZincBind considers biological assemblies, as described in PDB files, and discards zincs that have fewer than three liganding atoms (suggesting they are part of zinc salts), thus ensuring that the sites are biologically relevant. The web site allows the data to be downloaded, browsed and searched. Filters can be applied for structure quality and the data can also be accessed via REST web services for use in more complex analysis pipelines.

The dataset contained in this resource will be useful to researchers working on zinc binding site prediction, zinc binding site modelling and metalloprotein engineering.

The resource will be expanded in future with further structural and sequence annotations, such as binding site geometry, and with additional external database annotations. More advanced search tools are also planned, and the website will be enhanced as needed.

The web site may be accessed at zincbind.bioinf.org.uk and the code may be downloaded from github.com/samirelanduk/zincbind.

## Supplementary Material

Supplementary Data

## Notes

**Database URL:** zincbind.bioinf.org.uk
